# Renal Amyloidosis: A Clinicopathological Study From a Tertiary Care Hospital in Pakistan

**DOI:** 10.7759/cureus.21122

**Published:** 2022-01-11

**Authors:** Safina Ahmed, Humaira Nasir, Ambreen Moatasim, Fareeha Khalil

**Affiliations:** 1 Pathology, Shifa College of Medicine, Shifa Tameer-E-Millat University, Islamabad, PAK; 2 Histopathology, Shifa International Hospital, Islamabad, PAK; 3 Pathology, Shifa International Hospital, Islamabad, PAK; 4 Nephrology, Shifa International Hospital, Islamabad, PAK

**Keywords:** immunofluorescence, glomerular deposits, nephrotic syndrome, multiple myeloma, tuberculosis, etiology, renal biopsy, al amyloidosis, aa amyloidosis, renal amyloidosis

## Abstract

Introduction

Systemic amyloidosis can affect any organ in the body, but the kidney is the most commonly involved site. It is characterized by the extracellular deposition of insoluble fibrillar proteins. Amyloid deposits can be identified histologically by Congo red stain, which gives apple-green birefringence under polarized light. Typing of renal amyloidosis is done by direct immunofluorescence on frozen tissue. The most common types of amyloidosis seen in renal tissue are amyloid light chain (AL) primary amyloidosis and amyloid A (AA) secondary amyloidosis. Although primary amyloidosis is considered the most common type in western countries, however, in the subcontinent region, secondary amyloidosis is more common.

The spectrum of signs and symptoms in renal amyloidosis is variable including isolated proteinuria, nephrotic syndrome, hypertension, hypotension, and renal insufficiency. The present study aims to evaluate the incidence and aetiology of various types of renal amyloidosis, determine their distribution within the kidney, and study various clinicopathological features.

Objective

The present study aims to evaluate the aetiology and clinicopathological profile of renal amyloidosis, determine its various types, and their distribution within the kidney.

Materials and methods

This retrospective cross-sectional study was conducted from 1st January 2013 to 31st December 2020 at the Department of Histopathology, Shifa International Hospital (SIH), Islamabad. All renal biopsies diagnosed as renal amyloidosis were included in the study. Data were analysed using SPSS version 23 (IBM Corp., Armonk, NY). Frequency and percentages were calculated for qualitative variables, and mean and standard deviation were calculated for quantitative variables.

Results

A total of 131 cases were diagnosed with renal amyloidosis during the study period of eight years (from 1st January 2013 to 31st December 2020) at SIH. The age range varied from 17 to 82 years. The mean age of the patients was 45 ± 16.33 years. Out of 131 patients, 82 (62.6%) were males and 49 (37.4%) were females. Amongst them, 72 (54%) cases were diagnosed with secondary AA amyloidosis and 16 (12%) cases were diagnosed with primary AL amyloidosis. The rest of the cases 43 (34%) were of indeterminate type. The associated conditions in secondary amyloidosis were tuberculosis in 41 (57%), rheumatoid arthritis in 16 (22%), ankylosing spondylitis in five (7%), lymphoma in three (4%), diabetes in two (3%), and chronic osteomyelitis, chronic heart disease, hepatitis, and vasculitis in one case each (1.7%). Out of 16 cases reported with AL amyloidosis, 10 cases (62.5%) had a history of multiple myeloma. The most common clinical presentation was nephrotic syndrome followed by subnephrotic proteinuria, renal failure, and hypertension.

Conclusion

The findings of the present study show underlying etiological factors and clinicopathological characteristics of renal amyloidosis. AA amyloidosis is the most common type of renal amyloidosis in our study and tuberculosis is the most common aetiological factor. AL amyloidosis is less frequent in our population.

## Introduction

Systemic amyloidosis can affect any organ in the body, but the kidney is the most commonly involved site. It is characterized by the extracellular deposition of insoluble fibrillar proteins. The most common types of amyloidosis seen in renal tissue are amyloid light chain (AL) primary amyloidosis and amyloid A (AA) secondary amyloidosis [1,2]. Other uncommon types of amyloidosis that can affect the kidney are those derived from β 2-microglobulin (Ab2M) in dialysis-associated nephropathy and amyloid β protein (Ab) in Alzheimer’s disease and Down's syndrome. Other rare hereditary forms also occur, which include fibrinogen A alpha (AFib), apoprotein (Apo AI, AII, AIV), transthyretin (ATTR), lysozyme, and leukocyte chemotactic factor 2 (LECT2). Although primary amyloidosis is considered the most common type in western countries, secondary amyloidosis is more common in Pakistan and other Asian countries [3].

Amyloid deposits can be identified histologically by Congo red stain, which gives apple-green birefringence under polarized light [4]. Categorization of renal amyloidosis into primary or secondary is done by demonstrating clonality of light chains by using immunofluorescence (IF) and immunohistochemistry (IHC) for kappa, lambda light chains, and serum amyloid A (SAA) protein. Determining the type of renal amyloidosis is essential for treatment and prognosis [5].

The spectrum of signs and symptoms in renal amyloidosis is variable including isolated proteinuria, nephrotic syndrome, hypertension, hypotension, and renal insufficiency [6]. The present study aims to evaluate the incidence and aetiology of various types of renal amyloidosis and determine their distribution within the kidney and associated clinical manifestations.

## Materials and methods

This cross-sectional study was conducted from 1st January 2013 to 31st December 2020 at the Department of Histopathology, Shifa International Hospital (SIH), Islamabad after approval from the Institutional Review Board and Ethics Committee. All renal biopsies fulfilling adequacy criteria (at least 10 glomeruli for light microscopy [LM] and at least one for IF) and diagnosed as renal amyloidosis were included in the study (n = 131).

The patients’ demographic and clinical data were retrieved from case files. The clinical data included the presence of isolated proteinuria, nephrotic syndrome, hypertension, renal failure, plasma cell dyscrasias, and chronic inflammatory conditions. The histopathological data including LM and IF findings were taken from Laboratory Information System (LIS).

Clinical definitions

Nephrotic syndrome includes the following: nephrotic proteinuria (≥3.5 g/24 h), hypoalbuminemia (serum albumin ≤ 2.5 g/dL), hyperlipidaemia (>200 mg/dl), and edema [1]. Subnephrotic proteinuria refers to the protein level of 300 mg to 3.5 g/24 h [1]. Renal failure refers to serum creatinine level of >1.5 mg/dL [1]. Hypertension refers to systolic blood pressure (BP) ≥ 140 mmHg and/or diastolic BP ≥ 90 mmHg. Hypotension refers to systolic BP < 100 mmHg.

Histopathological analysis

At our hospital, two cores of the medical renal biopsy were obtained for pathologic evaluation. One core was processed for LM and fixed in 10% buffered formalin. The other core was kept fresh and snap-frozen for the IF study. All biopsies were examined by the consultant histopathologists in co-operation with nephrologists to arrive at the correct diagnosis.

Light Microscopic Evaluation

For LM, 10 serial sections were cut at a thickness of 2 um and stained by hematoxylin and eosin (H&E) and special stains, i.e., Masson's trichrome, periodic acid-Schiff, silver stain (Gomori's methenamine silver), and Congo red stain. Amyloid was visualized as amorphous eosinophilic extracellular material on H&E, which stained positive with Congo red and showed apple-green birefringence under polarizer. The amyloid deposits were examined for the dominant site of involvement, i.e., glomerular, interstitial, vascular, or all compartments.

Immunofluorescence

Tissue specimens for IF were snap-frozen in liquid nitrogen and cut on cryotome. The tissue was stained by the direct method using fluorescein isothiocyanate (FITC) conjugated antisera mono-specific for kappa and lambda along with immunoglobulin G (IgG), immunoglobulin A (IgA), immunoglobulin M (IgM), C3, and C1q. The slides were visualized under the fluorescence microscope.

Types of Amyloidosis

Amyloidosis is categorized as follows: AL amyloidosis (light-chain restriction using direct IF and evidence of monoclonal plasma cell proliferation disorder); AA amyloidosis (history of chronic inflammation and absence of light-chain restriction); and undetermined type (cases not fitting in the definition of AL or AA amyloidosis).

Statistical analysis

Data were analysed using SPSS version 23 (IBM Corp., Armonk, NY). Frequency and percentages were calculated for qualitative variables, and mean and standard deviation were calculated for quantitative variables.

## Results

**Figure 1:**
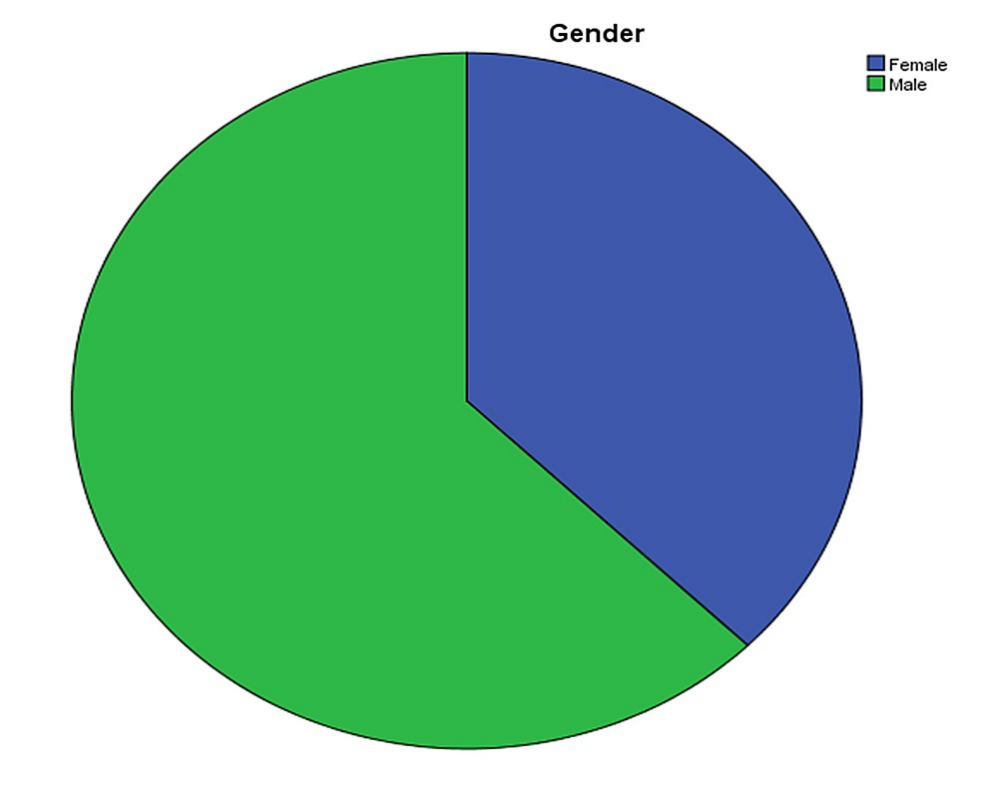
Percentage of male and female patients.

A total of 131 cases were diagnosed with renal amyloidosis during the study period of eight years (from 1st January 2013 to 31st December 2020) at SIH. These cases were retrieved from the archives of the pathology department. The age range varied from 17 to 82 years. The mean age of the patients was 45 ± 16.33 years. Out of 131 patients, 82 (62.6%) were males and 49 (37.4%) were females (Figure 1).

**Figure 2:**
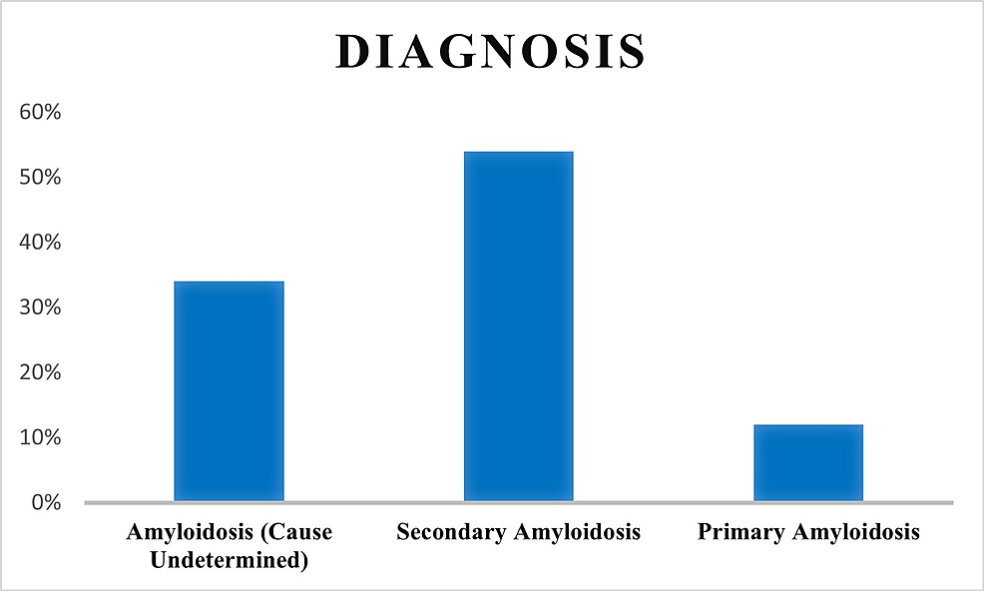
Bar graph depicting the percentage of types of amyloidosis.

Amongst 131 patients, 72 (54%) cases were diagnosed with secondary AA amyloidosis and 16 (12%) cases were diagnosed with primary AL amyloidosis. The rest of the cases (43, 34%) were of indeterminate type. The associated conditions in secondary amyloidosis were tuberculosis in 41 (57%), rheumatoid arthritis in 16 (22%), ankylosing spondylitis in five (7%), lymphoma in three (4%), diabetes in two (3%), and chronic osteomyelitis, chronic heart disease, hepatitis, and vasculitis in one case each (1.7%) (Figure 2). Out of 16 cases reported with AL amyloidosis, 10 cases (62.5%) had a history of multiple myeloma.

**Figure 3:**
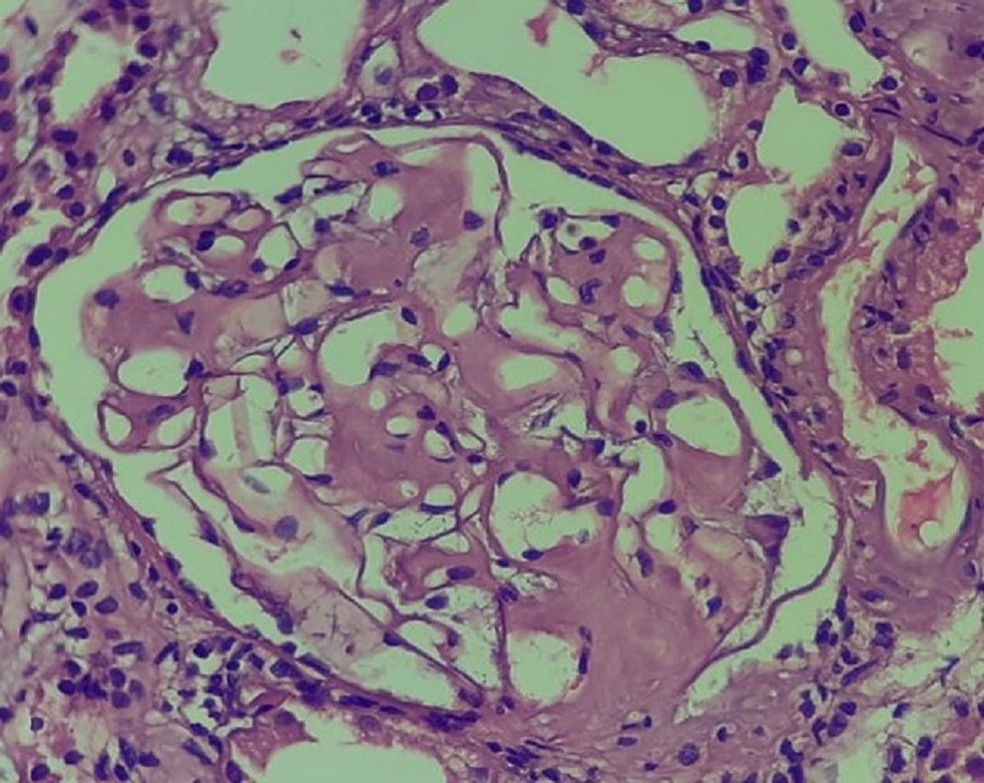
Mesangial glomerular involvement by amyloidosis (original magnification, 200x).

**Figure 4:**
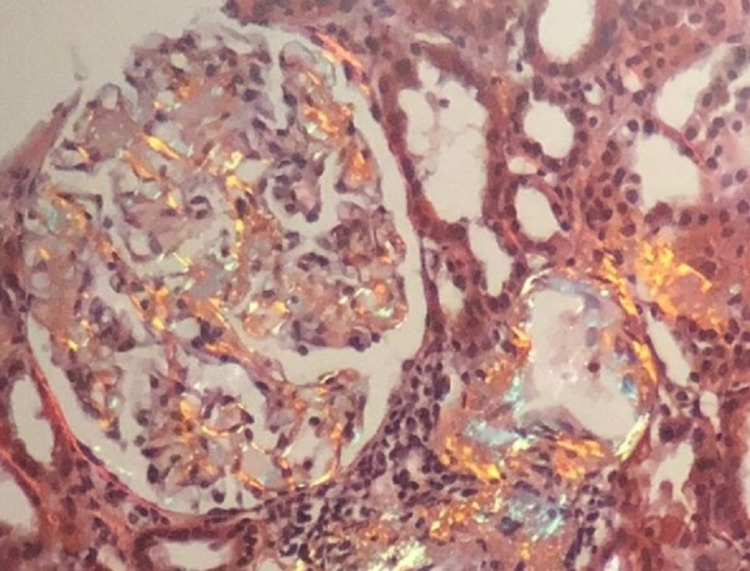
Congo red-stained section showing apple-green birefringence under polarized light (original magnification, 200x).

Out of 131 patients, 90 patients (68.7%) presented with nephrotic syndrome, 23 (17.6%) patients presented with renal failure, 17 (13%) patients presented with subnephrotic proteinuria, and one (0.8%) patient presented with hypertension. As for morphological findings, tubular atrophy was seen in 57% cases, interstitial fibrosis in 60% cases, and global glomerulosclerosis in 9% cases. A total of 115 (87%) cases showed pink homogenous amyloid deposits in glomeruli whereas 16 (13%) cases showed glomerular, tubular, and vascular deposits, which were positive on Congo red stain and showed apple-green birefringence under the polarized lens (Figures 3, 4). On IF, 43 (32%) cases displayed deposition of only mesangial IgM deposits, 78 (60%) cases showed deposition of mesangial IgA, IgG, and C3 ± C1q in addition to IgM, and 10 (8%) cases showed negative IF. Clinicopathological features of primary and secondary amyloidosis are shown in Table 1.

**Table 1:** Clinicopathological features and demographics of primary and secondary renal amyloidosis. AA, amyloid A; AL, amyloid light chain.

Parameters	Total patients	AA amyloidosis	AL amyloidosis	Amyloidosis (cause undetermined)
No. of patients	131(100%)	72 (54%)	16 (12%)	43 (34%)
Mean age	45 years	46 years	52 years	47 years
Gender (males)	82 (62%)	45 (55%)	12 (15%)	25 (30%)
Gender (females)	49 (37.4%)	27 (55%)	4 (8%)	18 (37%)
Nephrotic syndrome	90 (68%)	49 (37%)	13 (9%)	28 (21%)
Renal failure	23 (17%)	12 (9%)	3 (2%)	8 (6%)
Subnephrotic proteinuria	17 ((12%)	10 (7%)	0 (0%)	7 (5%)
Hypertension	1 (0.7%)	1 (0.7%)	0 (0%)	0 (0%)
Tubular atrophy	75 (57%)	41(31%)	9 (7%)	25 (19%)
Interstitial fibrosis	79 (60%)	42 (33%)	9 (7%)	28 (21%)
Global glomerulosclerosis	13 (9%)	7 (5%)	1 (0.7%)	5 (3.3%)

## Discussion

Renal disease is a common manifestation and a most important contributor to morbidity in patients with systemic amyloidosis. Only a few studies that report clinicopathological features of renal amyloidosis have been done in Pakistan. We are presenting our experience from a single centre, Shifa International Hospital, Islamabad, which is a tertiary care teaching hospital attached to a medical college and university. We report 131 cases of renal amyloidosis over eight years, which accounts for almost 1.6% of all the renal biopsies in the study period. A previous study done in Pakistan by Absar et al. in 2015 at Agha Khan Hospital Karachi, reported a 4% biopsy incidence of renal amyloidosis out of 435 native renal biopsies. Biopsy incidence of renal amyloidosis reported by an Indian study done in 2018 was 5.7% and an Egyptian study done in 2013 was 2.5% [7].

In our study, the mean age of the patients was 45 years. Patients with AL amyloidosis had a mean age of 52 years, which is higher than the mean age of patients with AA amyloidosis. Similarly, a study done by Kalle et al. reported a mean age of 35 years for AA amyloidosis and 54 years for AL amyloidosis [5]. It infers that AL amyloidosis occurs in relatively older age groups. In their study, Gupta et al. have mentioned that the median age of diagnosis of secondary amyloidosis is rising from 50 to 70 years due to effective treatment strategies available nowadays [8]. The male to female ratio reported in our study was 1.6:1 whereas, in a study done by Kalle et al., it was 2.3:1, which suggests male predominance [5].

In the present study, the majority (54%) of the cases were of secondary amyloidosis, which is similar to those reported in other Pakistani and Asian studies [9]. We reported the most common causes of secondary amyloidosis as tuberculosis, rheumatoid arthritis, ankylosing spondylitis, and other chronic inflammatory conditions. Similarly, previous studies show that chronic infections such as tuberculosis and leprosy are prevalent in India and developing Asian countries, which account for more cases of secondary amyloidosis [5]. Papa and Lachmann have also reported similar etiological factors for AA amyloidosis in their study [10]. A case study conducted by Tank et al. reported renal amyloidosis secondary to tuberculosis in the paediatric age group [11]. A study performed in Turkey suggested familial Mediterranean fever as the major cause of secondary amyloidosis [12].

On the contrary, primary amyloidosis is more common in the western world. AL amyloidosis is almost always associated with plasma cell or B-cell lymphoproliferative disorders. Monoclonal light chains are nephrotoxic and get deposited in kidneys leading to proteinuria [13,14]. In the current study, we reported 16 cases of AL amyloidosis with 62% having an association with multiple myeloma. A Turkish study reported 30 cases of AL amyloidosis amongst 190 cases of renal amyloidosis. Out of 30 cases, 8 (26%) had underlying multiple myeloma and two cases had plasma cell dyscrasias [15]. Kidney involvement occurs in approximately 70% of patients with AL amyloidosis. The risk of dialysis in patients who have either decreased estimated glomerular filtration rate (eGFR) or proteinuria is 11%-25% [16,17]. Therefore, early diagnosis is crucial to deliver effective therapy and prevent organ damage [18].

In our study, the most common clinical presentation was nephrotic range proteinuria (68%). Studies conducted by Dember and Kościelska et al. also stated proteinuria as the most common clinical presentation in patients with renal amyloidosis. It can range from subnephrotic to massive proteinuria, which is caused by predominant glomerular involvement [19,20]. Renal failure and hypertension are two other common presentations of these patients. Khalighi et al. reported renal failure as the second common clinical symptom after nephrotic range proteinuria [21]. Kuroda et al. have described a significant association between renal function and the area of amyloid deposition in the kidney [22]. In patients with AL amyloidosis, other organs may be involved such as the heart and peripheral nerves. These patients may present with postural hypotension, palpitations, dyspnea, and heart failure [23].

In the present study, 87% of cases showed pink homogenous amyloid deposits in the glomeruli only, whereas 13% showed deposits in glomeruli, tubules, and interstitium. Studies done by Min et al. and Sethi and Theis have reported glomerular deposition as the most common site of involvement followed by involvement of other compartments, i.e., tubules, interstitium, and/or vessels. In early presentations, only a few mesangial areas are involved and hence can be missed by routine histologic examination. Later, more extensive involvement of the mesangium occurs, which shows the nodular appearance and can simulate mesangial diabetic glomerulosclerosis [24,25].

Typing of amyloid is critical in the selection of appropriate treatment. AL amyloidosis is distinguished on IF by deposits that contain monoclonal immunoglobulin κ or the λ light chains. Thus, staining for these two proteins helps in differentiating AL from AA amyloidosis [26]. Several other methods are currently used, including IHC, mass spectrometry, and immunogold labelling for subtyping of amyloid. An IHC antibody panel to differentiate different amyloid proteins is considered a sensitive and reliable tool [27,28].

The treatment for secondary amyloidosis is to treat the underlying cause, i.e., treatment of tuberculosis by antituberculosis drugs. Chronic inflammatory processes like rheumatoid arthritis and ankylosing spondylitis are treated by anti-inflammatory drugs and immunosuppressive agents. The treatment of primary amyloidosis is to eradicate the underlying source of a clonal plasma cell population. Chemotherapeutic drugs and autologous stem cell transplantation are used for this purpose [29,30].

Our study has certain limitations. The IHC study at our centre was not routinely performed to identify the subtypes of amyloidosis.

## Conclusions

The findings of the present study show underlying etiological factors and clinicopathological characteristics of renal amyloidosis. AA amyloidosis is the most common type of renal amyloidosis in our study and tuberculosis is the most common etiological factor. AL amyloidosis is less frequent in our population.
